# 46,XY disorder of sex development due to 17-beta hydroxysteroid dehydrogenase type 3 deficiency: a plea for timely genetic testing

**DOI:** 10.1186/s13633-016-0030-x

**Published:** 2016-06-15

**Authors:** Chelsey Grimbly, Oana Caluseriu, Peter Metcalfe, Mary M. Jetha, Elizabeth T. Rosolowsky

**Affiliations:** Division of Endocrinology, Department of Pediatrics, University of Alberta, Edmonton Clinic Health Academy, 11405- 87th Ave., Edmonton, AB T6G 1C9 Canada; Department of Medical Genetics, University of Alberta, 8-39 Medical Sciences Building, 8613 114 St., Edmonton, AB T6G 2H7 Canada; Division of Pediatric Urology, Department of Pediatric Surgery, University of Alberta, 2C3.79 WC Mackenzie Health Sciences Centre, Edmonton, AB T6G 2R7 Canada

**Keywords:** 17β-hydroxysteroid dehydrogenase type 3 deficiency, Disorders of sex development, 46,XY undervirilization

## Abstract

**Background:**

17β-hydroxysteroid dehydrogenase type 3 (17βHSD3) deficiency is a rare cause of disorder of sex development (DSD) due to impaired conversion of androstenedione to testosterone. Traditionally, the diagnosis was determined by βHCG-stimulated ratios of testosterone:androstenedione < 0.8.

**Case presentation:**

An otherwise phenotypically female infant presented with bilateral inguinal masses and a 46,XY karyotype. βHCG stimulation (1500 IU IM for 2 days) suggested 17βHSD3 deficiency although androstenedione was only minimally stimulated (4.5 nmol/L to 5.4 nmol/L). Expedient genetic testing for the *HSD17B3* gene provided the unequivocal diagnosis.

**Conclusion:**

We advocate for urgent genetic testing in rare causes of DSD as indeterminate hormone results can delay diagnosis and prolong intervention.

## Background

Disorders of sex development (DSD) occur when there is discordance among chromosomal, hormonal, and phenotypic sex. They require prompt and timely diagnosis because certain etiologies can result in acute medical decompensation. Even in the absence of a medical emergency, ambiguous genitalia can present a social emergency to the parents when ascribing a sex to their newborn child. This can be further magnified if parents have already become accustomed to the child being a certain sex. The evaluation for the etiology of a DSD involves measurements of hormones with genetic testing historically reserved for confirmatory purposes. Hormonal assessments can direct genetic testing, but results may not be straightforward and can be influenced by a multitude of physiologic and practical factors. We present a case of a rare cause of 46, XY DSD where timely genetic testing resulted in a rapid diagnosis. The unequivocal result of genetic testing facilitated a more confident execution of a management and therapeutic plan.

## Case presentation

A healthy 1-month-old female was referred to Pediatric Endocrinology for bilateral inguinal masses. She was the product of a non-consanguineous conception between parents of English and English/German descent.

Physical examination revealed a healthy child with female external genitalia. She had prominence of the labial folds with palpable masses in the inguinal canals. There was a urogenital opening without clitoromegaly. Pelvic ultrasound demonstrated inguinal gonads and absence of uterus and ovaries.

Due to the presence of inguinal gonads but absence of Müllerian structures, investigations were pursued for causes of undervirilization. Cytogenetic analysis confirmed a normal 46,XY complement. Baseline Luteinizing Hormone, Follicle Stimulating Hormone, and cortisol concentrations measured at 6 weeks of age were 1.5 U/L, 1.6 U/L, and 621 nmol/L, respectively. Anti-Mullerian Hormone levels were appropriate for an infant male, implying the presence of functioning Sertoli cells. At 6 weeks of age, baseline androstenedione level was 4.5 nmol/L and testosterone 1.1 nmol/L. These levels in themselves were elevated, and the baseline testosterone:androstenedione (T:A) ratio of 0.2 hinted at the etiology of 17β-hydroxysteroid dehydrogenase type 3 (17βHSD3) deficiency. However, even during the mini-puberty of infancy, hormonal values of various causes of 46,XY undervirilization can significantly overlap. The patient subsequently underwent a short βHCG stimulation test 1500 IU daily for 2 days (Table 1). The diagnosis of 17β-hydroxysteroid dehydrogenase type 3 (17βHSD3) deficiency was further suspected based on the low stimulated T:A (testosterone 2.1 nmol/L, androstenedione 5.4 nmol/L, T:A 0.4). These results directed genetic testing for 17βHSD3 deficiency. At the same time, the minimally increased value of the stimulated androstenedione level compared to the baseline (5.4 and 4.5 nmol/L, respectively) called into question the adequacy of the stimulation testing. Other causes of 46,XY undervirilization, such as 5-alpha reductase deficiency, remained possible. Additionally, approval for genetic testing was not a given. Taking into consideration the parents’ desire to cement a diagnosis, a prolonged βHCG stimulation test (1500 IU twice weekly for 2 weeks) was undertaken when the baby was 18 weeks old (Table 1).

**Table 1:** Hormone Levels and Ratios with βHCG Stimulation (SI units)

	Short βHCG protocol^a^		Prolonged βHCG Protocol ^b^		Reference Ranges
	Baseline	Stimulated	Baseline	Stimulated	
Androstenedione (A)	4.5	5.4	1.7	20.5	< 3.0 nmol/L
Testosterone (T)	1.1	2.1	< 0.2	10.9	< 14 nmol/L
Dihydrotestosterone (DHT)	227.7	559	N/A	N/A	414–2933 pmol/L
T:A	0.2	0.4		0.5	> 0.8
T:DHT	4.8	3.7	N/A	N/A	> 10

Androstenedione (A) (RIA, DIAsource); Testosterone (T) (electrochemiluminescence, Roche); Dihydrotestosterone (DHT) (LC/MS/MS, Esoterix)

^a^1500 Units βHCG daily for two consecutive days at age 6 weeks. Stimulated levels were measured 24 h following the second injection

^b^1500 Units βHCG every other day (2 times per week) for 2 weeks at age 18 weeks. Stimulated levels were measured 24 h following the 3rd injection. The Prolonged βHCG Protocol was discontinued when the genetic result became available. The protocols were adapted from [12]

The evaluation and management of an infant born with a DSD should be conducted in interdisciplinary teams with experience in caring for these very rare conditions, including endocrinology, genetics, urology, social work, and clinical chemistry. At the same time, the particulars of the local socio-political and geographic environment dictate the distribution of medical resources and, in our case, the availability of genetic testing. While the prolonged βHCG stimulation test was transpiring, our multidisciplinary clinical team pursued genetic testing for 17βHSD3 deficiency as the most likely diagnosis on the basis of the clinical evaluation and initial low T:A. This effort involved actively petitioning for government funding to cover genetic testing as per routine in our medical jurisdiction when genetic testing is performed in a lab outside the province. We could not be assured funding for genetic testing and pursued prolonged βHCG testing during the limited time frame of neonatal puberty. The prolonged βHCG stimulation testing proved overwhelming for the family due to the multiple injections, cost of βHCG, and the practicalities of their having to travel back and forth from their remotely located home to the laboratory. In addition, the parents were committed to raising the baby as a girl and experienced considerable anxiety over the presence of testes and the delays in diagnosis.

DNA sequencing analysis for the *HSDB173* gene was conducted in a commercially available, FDA accredited laboratory (Prevention Genetics, Marshfield, WI, USA) by Sanger sequencing of the full coding regions of exons 1-11, as well as ~20 basepairs of flanking non-coding DNA on either side of each exon. This revealed a homozygous mutation, previously reported as pathogenic with complete loss of enzymatic activity (c.389 A > G) [1]. Genetic analysis of the parents revealed that both were heterozygous for the same mutation in the *HSD17B3* gene. Array CGH analysis was completed using the CytoSure TM ISCA 8x60K V2.0 Oligonucleotide array (Oxford Gene Technology) and showed normal dosage across the genome.

The genetic analysis was reported before the prolonged βHCG protocol was completed. Once funding was approved, the prolonged testing was discontinued. The cost of genetic testing was comparable to prolonged βHCG stimulation testing. With the genetic diagnosis, our team was able to provide focused, anticipatory guidance and allay many of the parents’ anxieties.

## Discussion

46,XY disorders of sex development (DSD) are uncommon and may stem from disorders of androgen synthesis in the adrenal glands or testes or disturbances in androgen action [2]. Making a timely diagnosis is important to prevent medical crises due to associated hormonal deficiencies. Identifying the etiology is also helpful in assigning a sex of rearing, predicting response to hormonal therapy in infancy, counselling about expected challenges at puberty, and guiding decisions regarding gonadectomy. However, a lack of diagnosis can occur in up to 50 % of cases with sexual ambiguity and a male karyotype [3].

Deficiency of 17βHSD3 is a rare cause of XY undervirilization affecting 1 in 147 000 live births [4]. This may be an underestimate as patients with 17βHSD3 deficiency can be incorrectly diagnosed with androgen insensitivity syndrome (AIS) [5]. The 17βHSD3 enzyme is present mainly in testicular tissue and converts the relatively weak androgen, androstenedione, to its potent metabolite, testosterone. There are at least 12 isoforms of 17βHSD present in organs including the liver, brain, and skin [6, 7]. Impairment of testosterone synthesis during fetal development results in undervirilization of male external genitalia. Although testosterone synthesis is insufficient, Anti-Müllerian Hormone production remains intact, leading to absence of internal Müllerian structures. The phenotypic spectrum ranges from normal-appearing female external genitalia to microphallus with hypospadias and variable degrees of genital ambiguity in between [7].

Assessment of basal hormone levels is typically the first step in diagnosis. Baseline androstenedione, testosterone, and dihydrotestosterone (DHT) levels and their ratios may help discriminate between 17βHSD3 deficiency and other causes of 46,XY DSD [4, 8]. However, considerable overlap in hormone levels has been shown [4, 5, 8, 9]. A T:A less than 0.8 was originally thought to be diagnostic of 17βHSD3 deficiency [4, 5]. This ratio only applies if there is an observed stimulation of androstenedione because low T:A can be seen in other defects in testosterone synthesis, including Leydig cell hypoplasia and testicular dysgenesis. In our case, while the baseline and stimulated levels and ratios suggested the possibility of 17βHSD3 deficiency, the baseline and stimulated levels did not differ significantly from one another; the T:A ratio of less than 0.8 could not by itself be used to diagnose 17βHSD3 deficiency. Unfortunately, there is no consensus as to the minimum threshold of androstenedione that reflects adequate βHCG stimulation. Variations in assays may, in part, underlie the overlap observed among hormone results.

Beta-HCG stimulation testing does not definitively diagnose 17βHSD3 deficiency nor distinguish it from other causes of 46,XY DSD. In patients with genetically-proven *HSD17B3* mutations, βHCG stimulation does not consistently stimulate androstenedione levels, making it challenging to interpret a T:A ratio [9, 10]. Other studies have demonstrated that the T:A ratio can be > 0.8 before and after βHCG stimulation in proven cases of 17βHSD3 deficiency; solely relying on T:A < 0.8 as a diagnostic criterion would have ruled out the diagnosis [4, 9, 10]. One report describes three related patients with stimulated T:A ratios of 0.5, 1.5 and 3.4, even though they shared the same *HSD17B3* mutation (homozygous S232L) [10]. In another study, a stimulated T:A ratio of < 0.8 falsely suggested 17βHSD3 deficiency in 4–6 % of patients with a confirmed diagnosis of complete or partial androgen insensitivity based on androgen binding studies and mutational analysis, and over half of the cases of testicular dysgenesis had a low T:A ratio [5, 11]. These studies provide evidence that the stimulated T:A ratio is not reliably diagnostic of 17βHSD3 deficiency. With our case, we pursued genetic testing for 17βHSD3 deficiency to reach an unequivocal diagnosis, given that neither baseline nor stimulated T:A ratios are absolutely reliable.

Failure of hormone testing to elucidate clearly the cause of XY undervirilization may relate to the variability and lack of consensus among βHCG stimulation protocols. The protocols differ in dose and duration, ranging from 500 to 1500 units per day and as long as 2 days to one month [4, 8, 12–15]. Evidently there is no clear consensus on duration or dose of βHCG, with most studies recommending an initial short course of βHCG followed by prolonged βHCG stimulation if there is an inadequate rise in precursors such as androstenedione or testosterone [12]. Our patient’s baseline precursors did not increase much following the short βHCG stimulation protocol. As a result, our patient underwent a prolonged stimulation protocol, and the androstenedione demonstrated a more convincing rise from 1.7 to 20.5 nmol/L with the T:A ratio remaining < 0.8.

The parental perspective and experience during investigations are essential considerations. In our case, the parents were committed to raising their 1-month-old infant as a girl, and they experienced significant emotional distress over the uncertainty of a diagnosis of 17βHSD3 deficiency. We debated the timing of gonadectomy as a previous literature review demonstrated that early orchiectomy resulted in 100 % retention of the female gender role while 54 % of patients changed to the male gender role if orchiectomy was delayed [16]. Delaying gonadectomy until puberty would provide the opportunity to observe whether the child was predisposed to a male gender identity but may also theoretically contribute to gender dysphoria [16]. However, these outcome data are limited and based on a small sample of patients. A review of published studies found that 39–64 % of female-assigned patients with 17βHSD3 deficiency underwent gender role changes [16, 17]. It is also important to decide on gonadectomy before puberty to prevent unintended virilization. This is highlighted in case reports where phenotypic females were diagnosed with 17βHSD3 deficiency at puberty after developing significant virilization. It is hypothesized that androstenedione is converted to testosterone by extra-testicular 17βHSD isoforms at puberty, and removal of the testes reduces the main source of androstenedione [18, 19]. Furthermore, there is a risk of gonadoblastoma, quoted as high as 28 % in some studies, and this risk should be factored into decisions on gonadectomy [7, 20]. The decision about sex of rearing should be made in light of the best possible prediction of future sexual function, virilization, and satisfaction with gender identity. These predictions are often only a best guess, further blurred if the etiology is in question.

Historically, genetic testing for rare causes of DSDs has been reserved for confirmatory purposes, guided by the results of hormonal testing. In our case, we had enough suspicion following the short-course βHCG stimulation testing to rationalize the request for genetic testing for 17βHSD3 deficiency as the most likely diagnosis. In the mean time, prolonged βHCG stimulation was initiated with the hopes of clarifying the diagnosis and in case genetic testing was denied. βHCG was not covered by insurance for the baby’s specific indication, and the parents were required to pay out-of-pocket for two vials. It required 3 extra medical appointments to receive the injections and 2 laboratory visits to draw the blood work. The cost of genetic testing was $780 USD (Prevention Genetics, Marshfield WI, USA). We advocate that this cost is acceptable and that earlier genetic testing could mitigate against the financial and human costs associated with sole reliance on equivocal, hormone-based investigations.

Genetic and molecular knowledge, research, and innovation are rapidly changing the way we investigate, diagnose, and treat medical conditions. Recent advancements in genetic testing have allowed for more cost-effective methods using gene panels to test for genetically heterogeneous Mendelian conditions [21]. Single-gene testing is preferred if, following clinical and laboratory evaluations, a specific diagnosis is likely, as demonstrated by our case. However, in many centres, including ours, genetic testing and confirmation are not readily accessible due to lack of testing facilities and/or prohibitive costs. These issues can also cause undue delay. Therefore, although the utility of genetic testing is well-appreciated, clinical use of these tests in an expedient manner is not yet optimally implemented. The intended ears for our plea belong not only to we who care for patients with DSD, but also to policy-makers, researchers, governments, and funding agencies, so that we may work together to improve access to these technologies.

## Conclusion

Sex assignment in an infant with a 46,XY disorder of sex development can be a social emergency because it requires urgent decision-making about the sex of rearing and considerations of potential fertility, the role of gonadectomy, and future gender identity. We contend that such decisions should not be made without a concerted effort to confirm the diagnosis. Many publications recommend βHCG stimulation to aid in the diagnosis, but βHCG-stimulated hormone results can be unreliable with overlap across diagnoses. The appropriate protocol for βHCG stimulation remains uncertain. It seems reasonable to try a short βHCG stimulation test to direct confirmatory genetic testing. With recent advancements in the field of clinical and molecular genetics, we advocate for a more prominent role for, and more expedient access to, urgent genetic testing to enable early and accurate diagnosis of rare DSDs.

## Consent

Written informed consent was obtained from the patient’s parents for publication of this case report. A copy of the written consent is available for review by the Editor-in-Chief of this journal.

## Abbreviations

17βHSD3: 17β-hydroxysteroid dehydrogenase type 3
A: androstenedione
DHT: dihydrotestosterone
DSD: disorders of sex development
T: testosterone
βHCG: beta human chorionic gonadotropin

## References

[1] Moghrabi N, Hughes IA, Dunaif A, Andersson S (1998). Deleterious missense mutations and silent polymoprhisms in the human 17beta-hydroxysteroid dehydrogenase 3 Gene (HSD17B3). J Clin Endocrinol Metab.

[2] Ahmed SF, Hughes IA (2002). The genetics of male undermasculinization. Clin Endocrinol.

[3] Morel Y, Rey R, Teinturier C, Nicolino M, Michel-Calemard L, Mowszowicz I, Jaubert F, Fellous M, Chaussain JL, Chatelain P, David M, Nihoul-Fékété C, Forest MG, Josso N (2002). Aetiological diagnosis of male sex ambiguity: a collaborative study. Eur J Pediatr.

[4] Boehmer AL, Brinkmann AO, Sandkuijl LA, Halley DJ, Niermeijer MF, Andersson S, de Jong FH, Kayserili H, de Vroede MA, Otten BJ, Rouwé CW, Mendonça BB, Rodrigues C, Bode HH, de Ruiter PE, Delemarre-van de Waal HA, Drop SL (1999). 17ß-hydroxysteroid dehydrogenase-3 deficiency: diagnosis, phenotypic variability, population genetics, and worldwide distribution of ancient and de novo mutations. J Clin Endocrinol Metab.

[5] Ahmed SF, Iqbal A, Hughes IA (2000). The testosterone:androstenedione ratio in male undermasculinization. Clin Endocrinol.

[6] Moeller G, Adamski J (2009). Integrated view on 17beta-hydroxysteroid dehydrogenases. Mol Cell Endocrinol.

[7] George MM, New MI, Ten S, Sultan C, Bhangoo A (2010). The clinical and molecular heterogeneity of 17βHSD-3 enzyme deficiency. Horm Res Paediatr.

[8] Maimoun L, Philibert P, Cammas B, Audran F, Bouchard P, Fenichel P, Cartigny M, Pienkowski C, Polak M, Skordis N, Mazen I, Ocal G, Berberoglu M, Reynaud R, Baumann C, Cabrol S, Simon D, Kayemba-Kay’s K, De Kerdanet M, Kurtz F, Leheup B, Heinrichs C, Tenoutasse S, Van Vliet G, Grüters A, Eunice M, Ammini AC, Hafez M, Hochberg Z, Einaudi S, Al Mawlawi H, Nuñez CJ, Servant N, Lumbroso S, Paris F, Sultan C (2011). Phenotypical, biological, and molecular heterogeneity of 5α-reductase deficiency: an extensive international experience of 55 patients. J Clin Endocrinol Metab.

[9] Khattab A, Yuen T, Yau M, Domenice S, Frade Costa EM, Diya K, Muhuri D, Pina CE, Nishi MY, Yang AC, de Medonça BB, New MI (2014). Pitfalls in hormonal diagnosis of 17β-hydroxysteroid dehydrogenase III deficiency. J Pediatr Endocrinol Metab.

[10] Lee YS, Kirk JM, Stanhope RG, Johnston DI, Harland S, Auchus RJ, Andersson S, Hughes IA (2007). Phenotypic variability in 17β-hydroxysteroid dehydrogenase-3 deficiency and diagnostic pitfalls. Clin Endocrinol.

[11] Ahmed SF, Cheng A, Dovey L, Hawkins JR, Martin H, Rowland J, Shimura N, Tait AD, Hughes IA (2000). Phenotypic features, androgen receptor binding, and mutational analysis in 278 clinical cases reported as androgen insensitivity syndrome. J Clin Endocrinol Metab.

[12] Ahmed SF, Achermann JC, Arlt W, Balen AH, Conway G, Edwards ZL, Elford S, Hughes IA, Izatt L, Krone N, Miles HL, O’Toole S, Perry L, Sanders C, Simmonds M, Wallace AM, Watt A, Willis D (2011). UK guidance on the initial evaluation of an infant or an adolescent with a suspected disorder of sex development. Clin Endocrinol.

[13] Douglas G, Axelrad ME, Brandt ML, Crabtree E, Dietrich JE, French S, Gunn S, Karaviti L, Lopez ME, Macias CG, McCullough LB, Suresh D, Sutton VR (2010). Consensus in guidelines for evaluation of DSD by the Texas Children’s Hospital Multidisciplinary Gender Medicine Team. Int J Pediatr Endocrinol.

[14] Paris F, Gaspari L, Philibert P, Maimoun L, Kalfa N, Sultan C (2012). Disorders of sex development: neonatal diagnosis and management. Endocr Dev.

[15] Chan AO, But BW, Lee CY, Lam YY, Ng KL, Tung JY, Kwan EY, Chan YK, Tsui TK, Lam AL, Tse WY, Cheung PT, Shek CC (2013). Diagnosis of 5α-reductase 2 deficiency: is measurement of dihydrotestosterone essential?. Clin Chem.

[16] Chuang J, Vallerie A, Breech L, Saal HM, Alam S, Crawford P, Rutter MM (2013). Complexities of gender assignment in 17β-hydroxysteroid dehydrogenase type 3deficiency. Is there a role for early orchiectomy?. Int J Pediatr Endocrinol.

[17] Cohen-Kettenis PT (2005). Gender change in 46, XY persons with 5α-reductase-2 deficiency and 17β-hydroxysteroid dehydrogenase-3 deficiency. Arch Sex Behav.

[18] Andersson S, Geissler WM, Wu L, Davis DL, Grumbach MM, New MI, Schwarz HP, Blethen SL, Mendonca BB, Bloise W, Witchel SF, Cutler GB, Griffen JE, Wilson JD, Russel DW (1996). Molecular genetics and pathophysiology of 17 beta-hydroxysteroid dehydrogenase 3 deficiency. J Clin Endocrinol Metab.

[19] Rosler A, Bélanger A, Labrie F (1992). Mechanisms of androgen production in male pseudohermaphroditism due to 17β-hydroxysteroid dehydrogenase deficiency. J Clin Endocrinol Metab.

[20] Lee PA, Houk CP, Ahmed SF, Hughes IA (2006). International consensus conference on intersex organized by the Lawson Wilkins Pediatric Endocrine Society and the European Society for Paediatric endocrinology: consensus statement on management of intersex disorders. Pediatrics.

[21] Xue Y, Ankala A, Wicox WR, Hegde MR (2015). Solving the molecular diagnostic testing conundrum for Mendelian disorders in the era of next-generation sequencing: single-gene, gene panel, or exome/genome sequencing. Genet Med.

